# Replicating Cardiovascular Condition-Birth Month Associations

**DOI:** 10.1038/srep33166

**Published:** 2016-09-14

**Authors:** Li Li, Mary Regina Boland, Riccardo Miotto, Nicholas P. Tatonetti, Joel T. Dudley

**Affiliations:** 1 Dept of Genetics and Genomic Sciences, Icahn School of Medicine at Mount Sinai, One Gustave L. Levy Place, Box 1498, New York, NY 10029, USA; 2 Icahn Institute for Genomics and Multiscale Biology, Icahn School of Medicine at Mount Sinai, One Gustave L. Levy Place, Box 1498, New York, NY 10029, USA; 3 Dept of Biomedical Informatics, Columbia University Medical Center, 622 West 168th Street, PH-20, New York, NY, USA; 4 Dept of Systems Biology, Columbia University Medical Center, 622 West 168th Street, PH-20, New York, NY, USA; 5 Dept of Medicine, Columbia University Medical Center, 622 West 168th Street, PH-20, New York, NY, USA; 6 Observational Health Data Sciences and Informatics, Columbia University Medical Center, 622 West 168th Street, PH-20, New York, NY, USA

## Abstract

Independent replication is vital for study findings drawn from Electronic Health Records (EHR). This replication study evaluates the relationship between seasonal effects at birth and lifetime cardiovascular condition risk. We performed a Season-wide Association Study on 1,169,599 patients from Mount Sinai Hospital (MSH) to compute phenome-wide associations between birth month and CVD. We then evaluated if seasonal patterns found at MSH matched those reported at Columbia University Medical Center. Coronary arteriosclerosis, essential hypertension, angina, and pre-infarction syndrome passed phenome-wide significance and their seasonal patterns matched those previously reported. Atrial fibrillation, cardiomyopathy, and chronic myocardial ischemia had consistent patterns but were not phenome-wide significant. We confirm that CVD risk peaks for those born in the late winter/early spring among the evaluated patient populations. The replication findings bolster evidence for a seasonal birth month effect in CVD. Further study is required to identify the environmental and developmental mechanisms.

External replication and validation are essential to medical research^1^,^2^. The importance of replication has been demonstrated multiple times in the field of genetics where ethnic biases and other unanticipated confounding have led to novel findings that were irreproducible at other research centers^2^. Methods that utilize data obtained from Electronic Health Records (EHRs), such as Phenome-Wide Association Studies (PheWAS)^3^ require particular scrutiny^4^,^5^,^6^ as EHR-derived phenotype data can exhibit many biases that can influence results^7^. These include biases originating from variation in data quality^8^, the health-care process, and documentation^7^. Robust informatics-based methods attempt to adjust for these biases. However, replication and validation of novel findings remain critical for building confidence in EHR-based findings.

Recently, two of the co-authors (Boland and Tatonetti) developed a method called SeaWAS: Season-Wide Association Study that reveals relationships between birth month (as a proxy for prenatal/perinatal seasonality) and lifetime disease risk^9^. The SeaWAS method identified several novel birth month-disease relationships from EHR data^9^. The initial SeaWAS findings were based on data obtained from a patient population at Columbia University Medical Center (CUMC) located in New York City (NYC). SeaWAS replicated several previously reported disease-birth month findings. Those findings include asthma^10^, attention deficit hyperactivity disorder^11^, and reproductive performance^12^. In this study we attempt to validate previously reported SeaWAS findings using EHR data obtained from another academic medical center within NYC. The rationale for choosing this population is that they are independent, but representative, of the original study population and were likely exposed to the same seasonal climate conditions^13^. Specifically, we applied the SeaWAS method to an independent EHR data set representing a clinical population of 1,169,599 patients from Mount Sinai Hospital (MSH) in New York City. We found evidence to replicate seven of nine birth month-dependent cardiovascular conditions reported from the original SeaWAS study. These findings build confidence in several associations between birth month and cardiovascular condition risk. They also suggest the importance of further investigation into potential mechanisms underlying the increased risk.

## Methods

### Population

To perform a proper replication study, we sought to follow the original SeaWAS study as closely as possible^9^. We used electronic medical record data from Mount Sinai Hospital (MSH) located in NYC. Both CUMC and MSH are urban medical centers located in NYC and we expect they both would be subject to similar climate conditions^13^. EHR data at MSH is represented using a different schema and data module than was used in the original SeaWAS study at CUMC. Therefore we mapped the locally obtained International Classification of Diseases, version 9 (ICD-9) codes used at MSH to the Systemized Nomenclature for Medicine-Clinical Terms (SNOMED-CT) using the mapping table from the CDM v.4^14^. Approval for this study was obtained from the Institutional Review Board at MSH.

We extracted all individuals born between 1926 and 2000 inclusive (1,169,599 patients) who were treated at MSH (between 1979–2015), demographics given in Table 1. The median age of the MSH population was 53 years (interquartile range, IQR: 36–66), which skews older than the original CUMC population (median = 38 years, IQR: 22–58). Race and ethnicity demographics are represented differently between the MSH and CUMC datasets. At MSH, Hispanic could be reported as a race or an ethnicity or both (Table 1). Differences in the way that race and ethnicity are collected can result in some differences^15^. Overall sex, race, and ethnicity distributions did not differ significantly between the two institutions (p > 0.05, Table 1).

### Replicating Cardiovascular Condition – Birth Month Associations

We are especially interested in cardiovascular condition – birth month associations because they represent the novel findings from the original SeaWAS study^9^. Also at both institutions (CUMC and MSH) essential hypertension (associated with birth month at CUMC) was the most prevalent disease, signifying the clinical importance of this association. Therefore, we selected only circulatory system conditions (as defined using the ICD-9 codes) that were present in both the MSH and CUMC datasets (i.e., having at least 1000 patients at both MSH and CUMC). This represented a set of 108 conditions.

We modified the SeaWAS algorithm obtained from the public domain (code available here: https://github.com/maryreginaboland/SeaWAS) to fit the database schema of MSH. We first performed a phenome-wide exploration of all birth month – disease associations for conditions with at least 1000 patients (1433 conditions). We employed the Benjamini-Hochberg method to correct for multiple hypotheses and control for the false discovery rate (FDR)^16^ similar to the original study.

Next, we performed a post-hoc analysis, using Pearson’s correlation, to compare the birth month - disease risk curves between the two institutions (CUMC, MSH). We assess statistical significance by comparing the actual correlation between the two institutions to an empirically derived null distribution. We generated empirical null distributions for each condition by randomizing the birth month–disease risk curve from MSH. We then computed the Pearson correlation for the random curve and CUMC’s birth month-disease risk curve. We repeated this procedure 1000 times producing 1000 random correlation results. We determined the empirically derived p-value as the proportion of random correlations greater than the actual correlation between true MSH data and true CUMC data divided by 1000. If this value is zero then the p-value is reported as “p < 0.001.”

To place our findings in the context of biological mechanisms deemed important in birth month associations, we obtained peak flu season data obtained from the Centers for Disease Prevention and Control (CDC) data on flu activity from 1982–83 through 2013–14^17^. We also compared the serum vitamin D levels reported in Meier *et al*. 2004^18^.

## Results

### Phenome-Wide SeaWAS Results at MSH

**Figure 1:**
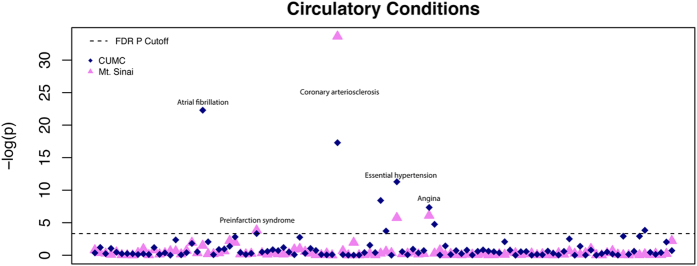
Manhattan Plot for Circulatory System Conditions for Mount Sinai (or MSH) and CUMC. Four conditions were found significant phenome-wide at both institutions: Coronary arteriosclerosis, Essential hypertension, Angina and Pre-infarction syndrome. Interestingly, Atrial fibrillation (most significant finding from CUMC) was not found to be significant phenome-wide at MSH.

We used SeaWAS to mine birth month associations at MSH for all 1433 conditions with at least 1000 individuals born between 1926 and 2000, inclusive. We applied multiplicity correction using FDR to adjust the p-values. We limited our analysis to only the circulatory system conditions (as defined by the ICD-9 codes) that were found in both datasets, allowing us to compare the patterns of birth month-disease risk between the two institutions. SeaWAS-CUMC analysis found 9 circulatory system conditions were associated with birth month at an FDR threshold of 0.05 computed phenome-wide (1433 conditions) (Fig. 1). Four of the circulatory disease associations were also found to be significant phenome-wide at MSH, namely, Coronary arteriosclerosis, Essential hypertension, Angina, and Pre-infarction syndrome.

### Pattern Analysis of Circulatory System Disease Risk – Birth Month Curves: MSH and CUMC

**Figure 2:**
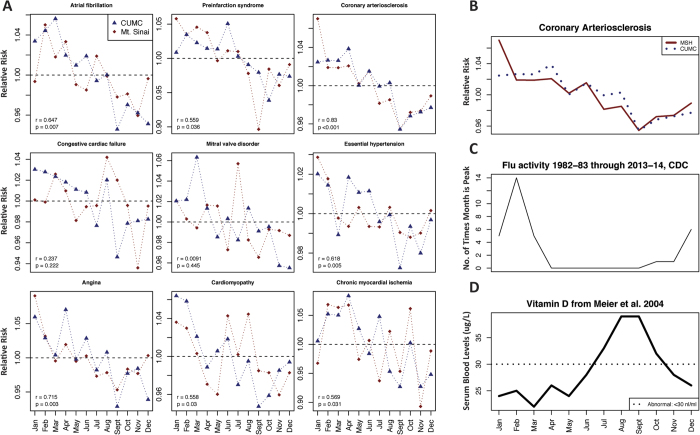
Cardiovascular Condition Risk vs. Birth Month Results from CUMC and MSH. (**A**) shows results from all nine cardiovascular conditions from both MSH (red line) and CUMC (blue line). Seven of nine cardiovascular conditions were correlated at a statistically significant level with MSH data (i.e., the birth month – condition patterns were correlated) using Pearson’s correlation. A significant pattern across the two institutions indicates that the birth month – condition relationship is the same. (**B**) shows the most correlated result between MSH and CUMC was coronary arteriosclerosis (r = 0.83, p < 0.001). (**C**) shows the comparison with the peak flu season month using CDC data on flu activity from 1982–83 through 2013–14 (URL: http://www.cdc.gov/flu/about/season/flu-season.htm). We also compared the serum vitamin D levels reported in Meier *et al*.^18^ in (**D**). We found that birth months that are also months with high serum vitamin D (Jul–Oct.) were ideal for lower coronary arteriosclerosis risk. Additionally, birth months with a high flu burden (Jan–Mar.) were high-risk birth months for coronary arteriosclerosis. This does not indicate that being born in flu season causes coronary arteriosclerosis later in life nor does it indicate that being born in a high vitamin D season lowers risk of coronary arteriosclerosis. These findings merely show support for proposed biological mechanisms, which require further validation from biologists.

We performed pattern analysis of the birth month – disease risk curves for the circulatory system conditions between MSH and CUMC (set of 108 conditions). This was done to ascertain whether or not the birth month – disease relationship was the same between the two institutions. We found that seven of nine CUMC findings had significantly similar patterns at MSH (Table 2). More detailed information on all 108 conditions can be found in the Supplemental File 1. The ICD-9 codes corresponding to each of the nine conditions in Table 2 are given in Supplemental File 2. In Fig. 2, we show the seasonal risk patterns at birth obtained from MSH and CUMC for all nine circulatory system conditions. Four of these nine circulatory system conditions were also significant at the phenome-wide level at MSH. Two associations, congestive cardiac failure and mitral valve disorder, did not have statistically significant patterns.

We overlaid data on seasonal variation in vitamin D levels and flu diagnosis levels to identify trends that could imply a biological rationale for observed cardiovascular – birth month association patterns. Figure 2D shows the seasonal variation in vitamin D levels^18^ along with the birth month – coronary arteriosclerosis risk curves from MSH and CUMC. Low vitamin D months correspond to high coronary arteriosclerosis risk birth months (Fig. 2B). In Fig. 2C, we also included data from the CDC (http://www.cdc.gov/flu/about/season/flu-season.htm) containing the number of times each month was the peak month for a given flu season. This data was aggregated from 1982–83 through 2013–14^17^.

## Discussion

Replication of novel research findings is critical to biomedical research^1^,^2^. Findings derived from EHRs are particularly important to replicate due to inherent biases present in EHR data^4^,^5^,^7^,^8^. For these reasons, we performed a phenome-wide SeaWAS using data from MSH to replicate previously reported associations between birth month and cardiovascular condition risk^9^. MSH is an ideal replication site as it shares climate and geography with the original site (CUMC)^13^. Seven of nine circulatory system conditions identified in the original SeaWAS-CUMC study had significantly correlated birth month – disease risk patterns. Additionally, four of these were significant at the phenome-wide level at MSH (Table 2).

### Replication of Main Cardiovascular Condition Findings Between MSH and CUMC Results

Coronary arteriosclerosis, essential hypertension, angina and pre-infarction syndrome were all significantly associated with birth month at the phenome-wide level at both MSH (after adjusting for 1433 tests) and CUMC (after adjusting for 1688 tests). Their seasonal patterns were also significantly correlated (Table 2, Fig. 2) supporting the connection between these conditions and birth month. While the patterns were highly correlated, in some instances the birth month with the max Relative Risk (RR) differed between the two institutions. For example, angina and coronary arteriosclerosis both experienced maximum RR’s in April using CUMC data versus January for the MSH data. However, the patterns for both of these conditions were highly correlated (coronary arteriosclerosis, r = 0.830; angina, r = 0.715). This underscores the importance of analyzing the entire pattern (Fig. 2) versus merely selecting maximum or minimum birth months to avoid misleading results.

Three conditions, atrial fibrillation, cardiomyopathy, and chronic myocardial ischemia, were significantly correlated between CUMC and MSH but were not significantly associated with birth month at the phenome-wide level at MSH. These conditions could be associated with birth month through their common comorbidities, namely essential hypertension, angina, coronary arteriosclerosis and pre-infarction syndrome^19^. However, it could also be the effect of sample size between the two institutions and the effects of stringent phenome-wide p-value adjustment. Many of these conditions had lower maximum RRs at MSH. For example, chronic myocardial ischemia had a maximum RR of 1.069 (0.973, 1.174) at MSH vs. 1.084 (1.011, 1.162) at CUMC. The significant correlation of the patterns between the two institutions supports the hypothesis of an underlying birth month effect for these conditions.

There are several additional reasons why results from CUMC and MSH may differ. Firstly, many of these circulatory system conditions are comorbid with one another (e.g., atrial fibrillation often occurs with other conditions such as essential hypertension)^19^. Conditions that replicated across institutions were ‘core’ heart conditions, such as essential hypertension, that often occur in the presence of other cardiac ailments^20^. These conditions may actually be driving the disease risk – birth month association. If this is the case, then they remain significant across institutions (provided the climate is the same). While both hospitals are located in NYC, they may serve patients with different socioeconomic demographics. These differences could play a role in the accessibility of certain healthcare options. Importantly, in spite of these issues, seven of nine CUMC circulatory system findings had statistically significant patterns at MSH^9^.

### Underlying Biological Mechanisms for Birth Month - Cardiovascular Condition Risk: Learning from Both MSH and CUMC

Several mechanisms have been proposed previously that could explain the relationship between cardiovascular conditions and birth month^9^. Vitamin D deficiency is known to increase cardiovascular disease risk, this is especially true for patients who already have essential hypertension^21^. Furthermore, vitamin D levels have been shown to vary seasonally in women^18^. Vitamin D levels in babies also depends on maternal vitamin D^22^. Another hypothesis to explain cardiovascular – birth month relationships is maternal flu infection. Researchers found that children born to survivors of the H1N1 1918 subtype had a >20% excess risk of cardiovascular disease later in life^23^. Maternal infection tends to be higher in the winter months (January–March) therefore this could contribute to increased risk among children born in those months.

Figure 2 shows that low vitamin D months correspond to high coronary arteriosclerosis risk birth months. However when vitamin D is low (Fig. 2D), the risk for flu infection is also high (Fig. 2C). It is unclear which mechanism (maternal flu infection and vitamin D) is potentially responsible for the birth month association. It could also be a combination of both mechanisms. Flu infection is high during the birth months with the highest risk, but vitamin D levels are also at their lowest during this same period (Fig. 2C,D). Also vitamin D plays a role in immune response^24^,^25^, which could indicate that peak flu season and low vitamin D occur together for a mechanistic reason (and are not independent of each other). Our confirmation of a link between cardiovascular conditions and birth month across two institutions – MSH and CUMC – in the same climate (NYC)^13^ supports the hypothesis that a biological mechanism tied to climate and seasonality can explain this increase in disease risk. Further research may elucidate whether vitamin D or flu infection, or a combination of the two, is contributing to the increase of cardiovascular condition risk by birth month.

### Limitations

A limitation of our approach includes the exclusive use of diagnosis codes collected during the clinical encounter and recorded in EHRs. Therefore, we know that these patients were diagnosed with a cardiovascular condition, but they may not necessarily have had cardiovascular disease. We used only diagnosis codes in order to properly validate the original results presented in Boland *et al*.^9^. Nine cardiovascular condition – birth month relationships were identified as significant in Boland *et al*.^9^. Only three of these nine conditions were diseases (Cardiomyopathy, Coronary Arteriosclerosis, and Mitral valve disorder), four were symptoms (Essential hypertension, Angina, Pre-infarction syndrome, Atrial fibrillation), one was indicative of an event (Chronic myocardial ischemia) and one was indicative of the patient state (Congestive cardiac failure). Importantly, we validated seven of nine cardiovascular conditions at MSH.

The condition most correlated between MSH and CUMC was coronary arteriosclerosis (Fig. 2B), which is a diagnosis for Coronary Artery Disease (r = 0.83, p < 0.001). Two conditions that failed to replicate were heart failure-related. Mitral valve disorder is a disease that can lead to heart failure and congestive cardiac failure is a diagnosis of the patient state indicating heart failure. We found that the conditions that validated across both sites were indicative of Coronary Artery Disease (coronary arteriosclerosis, chronic myocardial ischemia) and not heart failure.

## Conclusion

In this replication study, we study the cardiovascular condition – birth month relationship at another institution (MSH) with the same climate and urban setting as CUMC (both located in NYC). We found that seven of nine cardiovascular conditions associated with birth month at CUMC were also significantly correlated with the patterns revealed at MSH. These findings support the relationship between risk for cardiovascular conditions and birth month. We describe two leading putative mechanisms behind this relationship: maternal infection and low vitamin D levels. We also discuss the possibility of a combined mechanism (vitamin D is also involved in immune response) underlying the disease – birth month observations. Further study is required to identify the particular environmental and developmental mechanisms driving the observed associations.

## Additional Information

**How to cite this article**: Li, L. *et al*. Replicating Cardiovascular Condition-Birth Month Associations. *Sci. Rep.* **6**, 33166; doi: 10.1038/srep33166 (2016).

## Supplementary Material

Supplementary File 1

Supplementary File 2

## Figures and Tables

**Table 1:** Demographics of Patients Included in SeaWAS: CUMC and Mt Sinai.

Demographic	CUMC N (%), N = 1,749,400	Mt Sinai M (%),M = 1,169,599	P
**Sex**^1^			1.000
Female	956,465 (54.67%)	678,717 (58.03%)	
Male	791,534 (45.25%)	490,600 (41.95%)	
Other/Unidentified	1,401 (0.08%)	282 (0.02%)	
**Race**			0.603
White	665,366 (38.03%)	424,803 (36.32%)	
Other^1^	456,185 (26.08%)	165,423 (14.14%)	
Unidentified/Unknown	386, 533 (22.10%)	256,819 (21.96%)	
Black	189,123 (10.81%)	166,950 (14.27%)	
Declined	29,747 (1.70%)	NA	
Asian	20,746 (1.19%)	45,596 (3.90%)	
Native American/Indian	1,511 (0.09%)	2,447 (0.21%)	
Pacific Islander	189 (0.01%)	1,094 (0.09%)	
Hispanic/Latino	NA	106,467 (9.10%)	
**Ethnicity**			0.656
Non-Hispanic	590,386 (33.75%)	761,535 (65.11%)	
Unidentified	458,071 (26.18%)	208,899 (17.86%)	
Hispanic	361,123 (20.64%)	199,165 (17.03%)	
Declined	339,820 (19.42%)	NA	
**Other Attributes**		**Median (1**^**st**^**, 3**^**rd**^ **Quartile)**	
Total SNOMED-CT codes per patient	6 (1, 32)	7 (3, 22)	
Distinct SNOMED-CT codes per patient	3 (1, 8)	5 (2, 10)	
Age (year of service – year of birth)	38 (22, 58)	53* (36, 66)	
Treatment Year Range	1985–2013	1979–2015	

^1^Other (includes Hispanics not otherwise identified).

^*^Computed in days, age in years = age in days/365.25.

**Table 2:** Replication Results for Circulatory System Conditions Between MSH and CUMC: Phenome-Wide P-values and Pearson Correlation P-values.

Condition	Condition Type	Birth Month Risk - MSH		Birth Month Risk - CUMC		MSH	CUMC	MSH	CUMC	Pearson Corr.
		Low	High	Low	High	Max RR	Max RR	P*	P*	P**
Atrial fibrillation	Symptom	11	2	9	3	1.050	1.056	0.226	2.1 × 10^−^1^0^	0.007
**Coronary arteriosclerosis**	Disease	9	1	9	4	1.070	1.039	2.46 × 10^−15^	3.1 × 10^−8^	<0.001
**Essential hypertension**	Symptom	10	1	9	1	1.029	1.020	0.003	1.3 × 10^−5^	0.005
Congestive cardiac failure	State	11	8	9	1	1.042	1.030	0.760	2.2 × 10^−4^	0.222
**Angina**	Symptom	9	1	9	4	1.091	1.070	0.002	6.5 × 10^−4^	0.003
Cardiomyopathy	Disease	11	8	9	1	1.045	1.064	0.760	8.6 × 10^−3^	0.030
Chronic myocardial ischemia	Event	11	2	9	4	1.069	1.084	0.834	0.022	0.031
Mitral valve disorder	Disease	9	7	12	3	1.057	1.063	0.562	0.024	0.445
**Pre-infarction syndrome**	Symptom	9	1	10	6	1.058	1.051	0.022	0.036	0.036

^*^Phenome-Wide P-value, FDR adjusted (1688 conditions for CUMC, 1433 for Mt. Sinai).

^**^Empirically Derived P-value Using Random Permutations of Mt Sinai’s birth month distribution.

Bold indicates phenome-wide significance was attained at both CUMC and Mt. Sinai.
